# Diagnostic Accuracy of AdvanSure^TM^ and PowerChek^TM^ Real-Time PCR Assays for the Detection of *Mycobacterium tuberculosis* and Nontuberculous Mycobacteria

**DOI:** 10.3390/diagnostics15141776

**Published:** 2025-07-14

**Authors:** Johny Bajgai, Chi-Hyun Cho, Jong-Han Lee

**Affiliations:** 1 Department of Laboratory Medicine, Yonsei University Wonju College of Medicine, Wonju 26426, Republic of Korea; johnybajgai@yonsei.ac.kr; 2 Department of Convergence Medicine, Yonsei University Wonju College of Medicine, Wonju 26426, Republic of Korea; 3 Department of Laboratory Medicine, College of Medicine, Korea University Ansan Hospital, Danwon-gu, Ansan-si 15355, Republic of Korea; 4 Research Institute of Metabolism and Inflammation, Yonsei University Wonju College of Medicine, Wonju 26426, Republic of Korea

**Keywords:** *Mycobacterium tuberculosis*, nontuberculous mycobacteria, real-time PCR, culture diagnosis

## Abstract

**Background:** Accurate differentiation between *Mycobacterium tuberculosis* (MTB) and nontuberculous mycobacteria (NTM) is essential for proper diagnosis and treatment. This study compares the diagnostic performance of two commercial real-time PCR kits, AdvanSure^TM^ TB/NTM and Kogene PowerChek^TM^ MTB/NTM, for detecting MTB, NTM, and negative (no growth, NG) clinical specimens. **Methods:** A total of 390 clinical residual specimens were collected from patients between December 2022 and June 2023. The samples, including sputum, bronchoalveolar lavage, tracheal aspirate and body fluid, were initially tested with MGIT culture and then analyzed using both PCR kits. Sensitivity, specificity, positive predictive value (PPV), negative predictive value (NPV), and overall accuracy were evaluated. Discrepant results between the two PCR assays were further investigated using sequencing to identify the detected mycobacterial species, and final diagnoses were verified by culture results and review of electronic medical records. **Results:** Of the 390 specimens, both AdvanSure^TM^ and PowerChek^TM^ real-time PCR assays demonstrated 100% sensitivity for both MTB and NTM detection. For MTB detection, AdvanSure^TM^ demonstrated a specificity of 100%, with a PPV, NPV, and overall accuracy all reaching 100%. In comparison, PowerChek^TM^ showed a specificity of 98.62%, a PPV of 96.15%, an NPV of 100%, and an overall accuracy of 98.97%. For NTM detection, both AdvanSure^TM^ and PowerChek^TM^ exhibited identical performance metrics. The specificity was 99.58% for both assays, with a PPV of 99.34%, NPV of 100%, and an overall accuracy of 99.74%. Five discrepant results were finally confirmed as four NTM detection cases and one negative case by culture and clinical diagnosis which showed four cases of PowerChek^TM^ MTB+NTM detection and one case of NTM detection, respectively. **Conclusions:** The PowerChek^TM^ MTB/NTM real-time PCR kit demonstrated excellent diagnostic performance for the detection of MTB and NTM, with high sensitivity, specificity, and accuracy. Minor discrepancies, particularly in detecting MTB+NTM mixed infections, highlight the importance of complementary sequencing analysis for resolving uncertain results. These findings support the clinical utility of both PCR assays as reliable tools for rapid diagnosis of mycobacterial infections. PowerChek^TM^ showed occasional false positives, suggesting that optimizing the assay’s cutoff threshold or amplification parameters could enhance its specificity and reduce false-positive results in clinically ambiguous cases.

## 1. Introduction

Tuberculosis (TB) remains a major global health concern and continues to be the leading cause of death from a single infectious agent [1]. According to 2024 World Health Organization Global TB Report, an estimated 10.8 million people were diagnosed with TB worldwide, including 8.2 million newly diagnosed cases, the highest ever recorded [2]. Among them, the Republic of Korea has been particularly affected, maintaining the second highest TB incidence and fourth in the mortality rate among OECD member countries. As of 2023, South Korea reported 38.2 TB cases per 100,000 people, with a total of 19,540 affected individuals [3]. Given the persistent public health burden of *Mycobacterium tuberculosis* (MTB) and the increasing prevalence of nontuberculous mycobacteria (NTM) infections, rapid and accurate diagnostic methods are essential for effective disease management. Treatment approaches for MTB rely on standardized multi-drug regimens guided by well-established international guidelines, whereas NTM infections require species-specific, often prolonged and variable antibiotic combinations based on susceptibility testing. Delayed or misdiagnosis of MTB and NTM can lead to inappropriate treatment therapy and poor patient outcomes [4,5].

The MTB complex is the causative agent of TB, whereas NTM species have emerged as significant opportunistic pathogens, particularly among immunocompromised individuals and patients with chronic respiratory conditions [1,6]. Differentiating MTB from NTM is crucial, as their treatment approaches differ substantially, and misdiagnosis can lead to inappropriate therapy and public health consequences. Additionally, the detection of non-growing mycobacteria (NG) presents a considerable diagnostic challenge, necessitating more reliable and efficient methodologies [4,7]. Traditional diagnostic techniques, including acid-fast bacilli (AFB) staining and mycobacterial culture using the Mycobacteria Growth Indicator Tube (MGIT) system, remain the gold standard for Mycobacterium detection. However, these methods are time-consuming, requiring extended incubation periods that can delay clinical decision-making [8,9]. In contrast, molecular diagnostic techniques such as real-time polymerase chain reaction (PCR) have emerged as valuable tools for the rapid and precise detection of mycobacterial infections. These assays provide enhanced sensitivity, specificity, and faster turnaround times compared to culture-based methods [10,11,12]. Despite these advantages, the performance of real-time PCR assays varies based on assay design, target genes, and sample type, warranting thorough evaluation.

In Korea, real-time PCR assays targeting MTB and NTM have become widely adopted in clinical settings. Commercial real-time PCR tests were launched, such as AdvanSure TB/NTM Real-time PCR Kit (Invitros Co., Ltd., Cheongju-si, Republic of Korea), Real-Q M. tuberculosis Kit (Biosewoom, Seoul, Republic of Korea) and Anyplex MTB/NTM Real-time Detection (Seegene, Seoul, Republic of Korea). Additionally, the PowerChek MTB/NTM Real-time PCR Kit (Kogene Biotech, Seoul, Republic of Korea), introduced in 2013, enables the simultaneous detection of MTB and NTM. Notably, Anyplex and PowerChek are available for global use, further highlighting the clinical significance of these molecular tests [13]. Despite their widespread implementation, differences in diagnostic accuracy among these assays necessitate further evaluation to determine their clinical reliability and optimal application.

This study aims to evaluate and compare the diagnostic performance of two commercially available real-time PCR assay kits, the AdvanSure TB/NTM Real-time PCR Kit (Invitros Co., Ltd., Cheongju-si, Republic of Korea) and the PowerChek MTB/NTM Real-time PCR Kit (Kogene Biotech, Seoul, Republic of Korea) for the detection of MTB and NTM. MGIT culture results for MTB, NTM, and NG were used as the reference standards. By analyzing key parameters such as sensitivity, specificity, and diagnostic accuracy, this study aims to determine the clinical utility of these molecular assays in microbiology laboratories. The findings are expected to enhance diagnostic strategies for mycobacterial infections, facilitating timely and appropriate therapeutic interventions.

## 2. Methods

### 2.1. Sample Collection and Study Design

**Figure 1:**
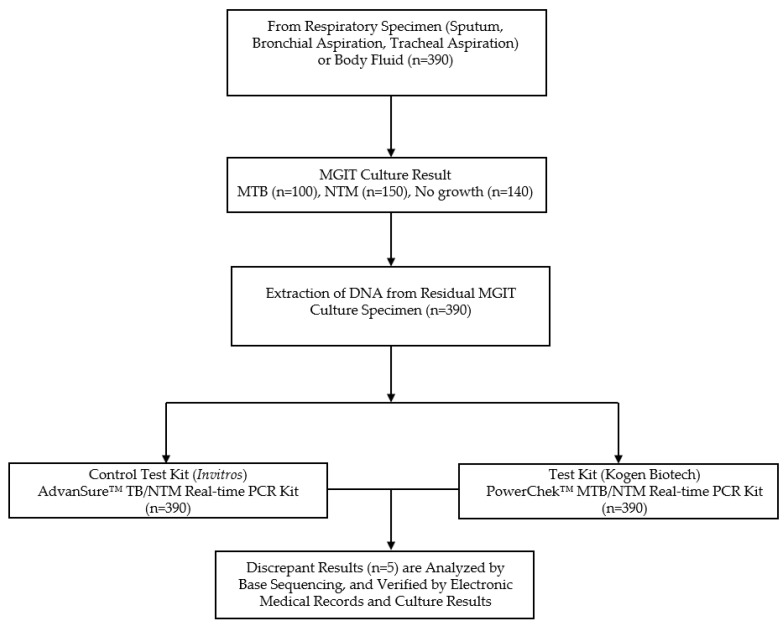
Study Design Flowchart. A total of 390 residual specimens (sputum, bronchial aspirates, tracheal aspirates, and body fluid) were analyzed using MGIT culture, and two real-time PCR assays—AdvanSure^TM^ TB/NTM (Reference) and PowerChek^TM^ MTB/NTM (Test). Specimens were classified into MTB-positive (*n* = 100), NTM-positive (*n* = 150), and NG (*n* = 140) groups. Five discrepant results between PCR assays were further analyzed by sequencing and verified by patients’ electronic medical chart review and culture results.

This study was conducted at the Department of Laboratory Medicine, Korea University Ansan Hospital, between December 2022 and June 2023. A total of 390 residual specimens were collected and analyzed, including sputum, bronchial aspirates, tracheal aspirates and body fluid. These residual specimens were the remaining portions of routine clinical samples left after initial diagnostic testing. The collected specimens were categorized as follows: 100 samples positive for MTB, 150 samples positive for NTM, and 140 negative samples/NG. All clinical samples were subjected to mycobacterial culture using the MGIT system, serving as the reference method. In parallel, two real-time PCR assays were used for molecular detection: the AdvanSure^TM^ TB/NTM Real-Time PCR Kit (Invitros Co., Ltd., Cheongju-si, Republic of Korea) served as the comparator (reference) assay, while the PowerChek^TM^ MTB/NTM Real-Time PCR Kit Ver. 1.0 (Kogene Biotech, Seoul, Republic of Korea) was evaluated as the test assay. Discrepant results between the two PCR assays were further investigated, using a base sequencing method to confirm the identity of the detected mycobacterial species, and verified with culture result and electronic medical chart review. All the samples were refrigerated for an extended period of time until the comparative study was performed. Repeated freeze–thaw cycles of clinical specimens were avoided to preserve sample integrity and prevent cross-contamination. This study was reviewed and approved by the Institutional Review Board of Korea University Ansan Hospital (IRB No. 2024AS0239; Dated: 24 September 2024). The study design flowchart is outlined in Figure 1.

### 2.2. Patient Classification

Patient classification in this study was performed using a composite diagnostic approach, incorporating clinical, microbiological, and molecular biological criteria, in accordance with the Korean Guidelines for the Diagnosis and Treatment of Tuberculosis and Non-tuberculous Mycobacterial Diseases. Patients were categorized into three groups based on microbiological, and clinical diagnostic findings:(1) MTB, (2) NTM, and (3) NG. All case classifications were verified using electronic medical records from the hospital.

### 2.3. Specimen Processing and MGIT Culture

A total of 390 residual specimens (sputum, bronchial aspirates, tracheal aspirates, and body fluid) were collected and processed using the BACTEC^TM^ MGIT^TM^ 960 system (BD, Sparks, MD, USA) according to the manufacturer′s instructions. Specimens were decontaminated using the Liquillizer–sodium hydroxide (NaOH) method (2% final concentration) (MetaSystems Indigo GmbH, Altlussheim, Germany), centrifuged at 3000× *g* for 20 min, and the resulting pellets were resuspended and inoculated into MGIT tubes (Becton, Dickinson and Company, Franklin Lakes, NJ, USA) containing Middlebrook 7H9 broth supplemented with oleic acid-albumin-dextrose-catalase (OADC) enrichment and polymyxin B–amphotericin B–nalidixic acid–trimethoprim–azlocillin (PANTA) antibiotic mixture. Simultaneously, specimens were inoculated onto solid Ogawa medium (3% agar) (Shin Yang Chemical Co., Ltd., Seoul, Republic of Korea).

Liquid cultures (MGIT 960) were incubated at 37 °C and monitored for up to 6 wks, while solid cultures (Ogawa media) were incubated for up to 8 wks. Positive MGIT cultures were confirmed for the presence of acid-fast bacilli (AFB) by auramine–rhodamine fluorescent staining (ELITechGroup Inc., Logan, UT, USA), followed by Ziehl–Neelsen staining (ELITechGroup Inc., Logan, Ut, USA) for confirmation. Smear results were graded from 1+ to 4+, with grades ≥1+ defined as smear-positive. Species identification was performed using a molecular method and an MPT64 antigen detection assay (immunochromatographic method) to differentiate MTB from NTM.

### 2.4. Evaluation of AdvanSure^TM^ TB/NTM Real-Time PCR and PowerChek^TM^ MTB/NTM Real-Time PCR Assay Kit

All clinical specimens were tested using two real-time PCR assays for comparative evaluation: the AdvanSure^TM^ TB/NTM Real-Time PCR Kit (Invitros Co., Ltd., Cheongju-si, Republic of Korea) and the PowerChek^TM^ MTB/NTM Real-Time PCR Kit Ver. 1.0 (Kogene Biotech, Seoul, Republic of Korea). For the AdvanSure^TM^ TB/NTM Real-Time PCR Kit, DNA extraction was performed on clinical samples following the manufacturer′s protocol. Specimens were initially treated with pre-treatment solutions, followed by incubation, centrifugation, and resuspension. DNA was extracted by heating with an extraction buffer at 100 °C for 20 min, then centrifuged at 13,000 rpm for 3 min. Subsequently, 5 μL of the clarified supernatant was transferred into the PCR reaction mix. For each PCR reaction (20 μL), the following components were used: 10 μL of 2X PCR mixture, 5 μL of primer/probe mix (targeting IS6110 for MTB, ITS gene for NTM, and the internal control (IC)), and 5 μL of extracted DNA. The amplification was performed using an SLAN-96P thermal cycler (Shanghai Lianchuan Bio-Tech Co., Ltd., Shanghai, China) under the following conditions: initial denaturation at 95 °C for 10 min, followed by 35 cycles of denaturation at 95 °C for 10 s, and annealing/extension at 62 °C for 40 s. The amplification targets included the IS6110 sequence (specific to MTB) detected in the FAM channel, and the ITS gene region (specific to NTM) detected in the HEX channel. The results were interpreted based on the threshold cycle (Ct) values according to the manufacturer’s instructions. Positive controls yielded Ct values between 21.0 and 24.0 for MTB, 22.5 and 25.5 for NTM, and 25.0 and 30.0 for the IC. Negative controls showed no Ct values for MTB+NTM, with the internal control Ct ≤ 30.0.

Moreover, for PowerChek^TM^ MTB/NTM Real-Time PCR Kit Ver. 1.0 (Kogene Biotech, Seoul, Republic of Korea) assay evaluation, samples were pretreated with 2% Liquillizer-NaOH and phosphate-buffered saline (PBS) for decontamination, followed by DNA extraction using the PowerPrep^TM^ TB DNA Extraction Kit, with the process validated by inclusion of a DNA process control (DPC). The extracted DNA (5 µL) was subjected to a quadruplex real-time PCR assay, which simultaneously detects MTB complex and NTM by targeting the ITS region (*Mycobacterium* genus-specific), IS6110 (MTB-specific), IC, and the DPC. Each 20 µL reaction contained 15 µL of premix (primers, probes, dNTPs, UDG, MgCl_2_, and Taq DNA polymerase). Amplification was performed on a CFX96^TM^ Dx Real-Time PCR System (Bio-Rad Laboratories, Hercules, CA, USA) with the following cycling conditions: 50 °C for 2 min (UDG activation), 95 °C for 5 min (polymerase activation), followed by 40 cycles of 95 °C for 5 s and 60 °C for 30 s. Fluorescence signals were recorded in the FAM (ITS), VIC (IS6110), ROX (IC), and Cy5 (DPC) channels. Validity of each PCR run was confirmed by control criteria: positive control (PC) Ct values of 23 ± 3 for all targets; negative control (NC) showing no amplification or Ct > 35 for ITS or not detected, >38 for IS6110 or not detected, and 23 ± 3 for IC, with DPC Ct > 33 or not detected; and negative process control (NPC) with no amplification or Ct > 35 or not detected for ITS, >38 or not detected for IS6110, IC Ct 23 ± 3, and DPC Ct 27 ± 3. Sample results were interpreted as MTB-positive when IS6110 Ct ≤ 38 with valid IC and DPC signals, NTM-positive when ITS Ct ≤ 35 with IS6110 negative, and negative when both ITS and IS6110 were undetected or above thresholds, provided IC and DPC Ct values were ≤33. The IC served to monitor PCR inhibition, while the DPC ensured successful nucleic acid extraction.

Discrepant results between the two assays were analyzed by base sequencing and further verified by reviewing patients’ electronic medical records.

### 2.5. Comparison of AdvanSure^TM^ TB/NTM Real-Time PCR and PowerChek^TM^ MTB/NTM Real-Time PCR Kit Detection Methods

The comparative analysis of two commercially available real-time PCR platforms; AdvanSure^TM^ TB/NTM Real-Time PCR Assay and PowerChek^TM^ MTB/NTM Real-Time PCR Kit Ver.1.0 based on key performance parameters for detection of MTB/NTM is shown in Table 1.

### 2.6. Base Sequence Analysis Method for Discrepant Results

Base sequence analysis was conducted on samples with discrepant results between culture, AdvanSure^TM^ TB/NTM Real-Time PCR Kit, and the PowerChek MTB/NTM Real-time PCR Kit. PCR amplification targeted the Hsp65 gene for NTM and the IS6110 gene specific to MTB, using primers described by a previously conducted study by Bensi and colleagues [14]. Amplified products (439 bp for Hsp65 and 550 bp for IS6110) were verified by agarose gel electrophoresis and subsequently sequenced. The PCR reactions for Hsp65 and IS6110 sequence analysis were conducted using a SimpliAmp Thermal Cycler (Thermo Fisher Scientific, Inc., Waltham, MA, USA) under the following conditions. For the Hsp65 sequence, an initial denaturation step was carried out at 95 °C for 10 min, followed by 40 cycles of denaturation at 95 °C for 30 s, annealing at 60 °C for 30 s, and extension at 72 °C for 30 s. A final extension was performed at 72 °C for 10 min. For the IS6110 sequence, the reaction began with an initial denaturation at 50 °C for 2 min and 95 °C for 10 min. Subsequently, 45 cycles were performed, including denaturation at 95 °C for 30 s, annealing at 65 °C for 30 s, and extension at 72 °C for 30 s. The final extension was carried out at 72 °C for 10 min. Sequence data were analyzed accurately to identify mycobacterial species, enabling confirmation of MTB and differentiation of NTM species in samples with conflicting diagnostic outcomes.

### 2.7. Data Processing and Statistical Analysis

The diagnostic performance of the two real-time PCR assays was evaluated by calculating sensitivity (the proportion of true positive samples correctly identified), specificity (the proportion of true negative samples correctly identified), positive predictive value (PPV; the proportion of positive test results that were true positives), negative predictive value (NPV; the proportion of negative test results that were true negatives), and overall accuracy (the proportion of correctly classified samples, including true positives and true negatives, among all samples tested). Each metric was presented with corresponding 95% confidence intervals (CIs), which were estimated using the exact Clopper–Pearson method. These calculations were based on 2 × 2 contingency tables comparing assay results against the composite reference standard defined in this study. All analyses were performed using an online diagnostic test evaluation calculator (MedCalc Statistical Software, https://www.medcalc.org/calc/diagnostic_test.php (Version 23.2.8; accessed 19 May 2025)). Concordance between the two PCR assays was evaluated, and discrepant cases were analyzed separately.

## 3. Results

### 3.1. Diagnostic Performance of AdvanSure^TM^ and PowerChek^TM^ Real-Time PCR Kits

The diagnostic performance of the AdvanSure^TM^ and PowerChek^TM^ PCR kits was evaluated for the detection of MTB, NTM, and NG samples. Both kits demonstrated exceptional sensitivity, specificity, and accuracy across all target groups. For instance, both AdvanSure^TM^ and PowerChek^TM^ real-time PCR assays demonstrated 100% sensitivity for both MTB and NTM. For MTB detection, AdvanSure^TM^ demonstrated a specificity of 100%, with a PPV, NPV, and overall accuracy all reaching 100%. In comparison, PowerChek^TM^ showed a specificity of 98.62%, a PPV of 96.15%, an NPV of 100%, and an overall accuracy of 98.97%. For NTM detection, both AdvanSure^TM^ and PowerChek^TM^ exhibited identical performance metrics. The specificity was 99.58% for both assays, with a PPV of 99.34%, NPV of 100%, and an overall accuracy of 99.74% (Table 2). These findings, supported by high 95% CIs, highlight the potential of both kits as effective diagnostic tools in clinical settings.

### 3.2. Resolution of Discrepant Results Between AdvanSure^TM^ and PowerChek^TM^ MTB/NTM Real-Time PCR Kits Using Base Sequence Analysis

Discrepant results between the AdvanSure^TM^ TB/NTM and the PowerChek^TM^ MTB/NTM Real-Time PCR Kits are presented in Table 3. Among 150 culture-confirmed NTM cases, the AdvanSure^TM^ kit identified all as NTM-positive cases, while the PowerChek^TM^ kit detected MTB+NTM in four cases (NTM010, NTM013, NTM117, and NTM135). Sequence analysis confirmed the presence of NTM in cases NTM010, NTM013, and NTM135 and MTB+NTM infection in NTM117, indicating partial concordance with the PowerChek^TM^ results. In addition, in one culture-negative case (NG035), both kits produced positive results for NTM, confirmed by sequencing, suggesting a possible limitation in culture sensitivity rather than PCR false positivity. Overall, these findings highlight that while both kits demonstrated high concordance with culture results for the detection of NTM, the PowerChek^TM^ kit showed a higher detection rate of MTB+NTM cases, potentially increasing its diagnostic sensitivity for mixed infections. However, discrepancies in the results emphasize the need for careful interpretation, particularly in cases with MTB+NTM or culture-negative findings, and support the complementary role of sequence analysis in resolving diagnostic inconsistencies. Ultimately, final diagnosis is made based on culture results with electronic medical chart review.

## 4. Discussion

The increasing frequency of NTM isolation from patients suspected of tuberculosis has raised concerns about the accurate diagnosis of mycobacterial infections [15,16,17]. Differentiating between MTB complex and NTM is critical, as their therapeutic regimens differ significantly. Incorrect diagnosis, whether through misidentification or lack of proper differentiation, could lead to the administration of inappropriate treatment, thereby compromising patient outcomes and contributing to unnecessary adverse effects [17,18,19]. Therefore, a precise and timely identification of mycobacteria is necessary for ensuring effective patient management.

Mycobacterial infections, particularly those caused by MTB and NTM, are not only a major public health burden globally but are further complicated by multiple factors such as microbial virulence, resistance profiles, host comorbidities, and environmental influences [20,21,22]. In addition, socio-economic disparities, migration patterns, and access to healthcare exacerbate the spread and impact of these infections. Despite global recognition of the threat posed by these pathogens, traditional diagnostic methods such as culture and biochemical testing remain time-consuming, technically demanding, and often require specialized laboratory infrastructure and personnel [6,22]. These limitations contribute to diagnostic delays, which are particularly concerning when rapid and effective treatment is necessary to control the progression of infection. In addition, the accurate identification of MTB and NTM is particularly critical, as the therapeutic approaches for tuberculosis differ from those for mycobacteriosis, and there are notable variations in antibiotic susceptibility among NTM species. Misidentification of these pathogens can result in inappropriate treatment, threatening patient outcomes and fostering the development of antimicrobial resistance [22]. Hence, the need for rapid, accurate diagnostic tools is imperative to ensure effective treatment and improve patient prognosis.

In response to these challenges, molecular diagnostic techniques, particularly real-time PCR, have revolutionized the diagnostic landscape for mycobacterial infections [11,13,23]. PCR assays, such as the AdvanSure^TM^ TB/NTM Real-Time PCR kit and the PowerChek^TM^ MTB/NTM Real-Time PCR kit Ver.1.0 evaluated in this study, have demonstrated their potential to substantially improve diagnostic efficiency by enabling rapid identification of MTB and NTM infections with high sensitivity and specificity. These kits are capable of distinguishing between MTB and NTM within hours, significantly reducing the time to diagnosis compared to conventional methods [12,13]. Our study findings support the high diagnostic performance of both PCR assays. Both AdvanSure^TM^ and PowerChek^TM^ real-time PCR assays demonstrated 100% sensitivity for both MTB and NTM. For MTB detection, AdvanSure^TM^ demonstrated a specificity of 100%, with PPV, NPV, and overall accuracy all reaching 100%. In comparison, PowerChek^TM^ showed a specificity of 98.62%, a PPV of 96.15%, an NPV of 100%, and an overall accuracy of 99.74%. For NTM detection, both AdvanSure^TM^ and PowerChek^TM^ exhibited identical performance metrics. The specificity was 99.58% for both assays, with a PPV of 99.34%, an NPV of 100%, and an overall accuracy of 99.74%. This result is consistent with previous studies that have highlighted the potential of PCR assays to not only detect single infections but also identify mixed infections, thereby improving the overall sensitivity of mycobacterial detection [13,24].

Several other reports have evaluated the clinical performance of commercial PCR kits for MTB and NTM detection, reporting variable results. For instance, Anyplex^TM^ MTB/NTM Real-Time Detection V2.0 (Seegene, Seoul, Republic of Korea) has shown MTB sensitivity ranging from 71 to 86% and specificity from 94.9 to 99%, while NTM sensitivity ranged from 44.9% to 100% and specificity from 97.7% to 97% [25,26,27,28]. Our results align with the findings on the high sensitivity of PCR kits for MTB detection, as both the Viasure^TM^ and Sansure^TM^ kits exhibited 100% sensitivity for MTB complex identification, consistent with the performance compared to gold standard methods. However, co-infection cases were not detected [22]. In our study, both PCR assays demonstrated excellent sensitivity; however, an occasional false-positive result was observed, highlighting the need to optimize assay parameters such as cutoff thresholds or amplification cycles to minimize false-positive results. While sensitivity remained high, refining these parameters could further enhance specificity, addressing a common challenge in molecular diagnostics that requires careful calibration. Importantly, our findings also showed that PCR could detect MTB infections in samples that were culture-positive for NTM only, highlighting the superior sensitivity of molecular techniques compared to conventional culture methods. This suggest that PCR can serve as a valuable supplementary tool to enhance the detection rates of mycobacterial infections, though confirmatory testing remains essential for accurate diagnosis [12,22]. Furthermore, identifying mixed MTB+NTM infections through PCR assays has significant clinical relevance, as it informs appropriate antimicrobial therapy and improves patient management.

Despite these promising results, a discordance rate of 1.28% (5 out of 390 cases) was observed. In these discrepant cases, the PowerChek^TM^ assay reported four MTB+NTM cases and one NTM-only infection, whereas the AdvanSure^TM^ assay identified five NTM-only infections. In order to resolve these discrepancies, gene sequencing was performed, which confirmed the presence of NTM in four of the five cases, with one case being an MTB+NTM mixed infection. Additionally, a comprehensive review of the patients’ electronic clinical records, including radiologic findings, treatment response, and risk factors, supported the sequencing results, upon which the final interpretation was made. These discrepancies may stem from differences in the target gene regions and primer/probe designs between the two assays. PowerChek^TM^ targets the IS6110 region for MTB and the hsp65 region for NTM, while AdvanSure^TM^ targets the IS6110 gene for MTB and the ITS gene region (specific to NTM), which may affect sensitivity in detecting low-copy or mixed infections. Similar discordant results due to assay design variability have been reported in previous studies comparing multiple PCR platforms for mycobacterial detection [1,29]. This finding highlights the importance of performing confirmatory tests such as sequencing in cases where PCR results indicate possible co-infections or conflict with clinical presentation or culture results. Importantly, these discrepancies have meaningful implications for clinical decision making and patient management in real-world settings. False-positive molecular results could potentially lead to an unnecessary initiation of anti-tuberculosis therapy, additional invasive diagnostic procedures, increased healthcare costs, and an increased psychological burden for patients. Overdiagnosis also risks contributing to anti-microbial resistance if treatment is misapplied. Conversely, false negatives or failure to detect co-infections could delay appropriate therapy and worsen patient outcomes [30,31]. These findings emphasize the necessity of integrating molecular results with clinical, radiological, and, whenever possible, culture-based or sequencing confirmation to achieve accurate diagnosis and guide optimal patient management. Although sequencing is considered the reference standard for distinguishing MTB and NTM due to its high specificity and detailed strain-level resolution, its routine use in clinical laboratories is limited by its higher cost, longer turnaround time, and the need for specialized expertise [32,33]. Therefore, in practical settings, sequencing is mainly used as a confirmatory test to validate and support discordant PCR results and mixed infection when rapid tests are inconclusive or insufficient.

While our study provides valuable insights into the comparative diagnostic performance of the PowerChek^TM^ and AdvanSure^TM^ assays, it is important to acknowledge several limitations. First, we used residual, clinical specimens rather than freshly collected samples. While this approach allows retrospective analysis, the use of residual clinical specimens stored under refrigeration for an extended period of time may introduce DNA degradation and potentially bias sensitivity estimates compared to fresh samples. Therefore, future studies employing prospectively collected, fresh specimens under standardized handling conditions are recommended to validate these findings. Second, the study adopted a case-control design by selecting samples with known MGIT culture results for the PCR assays’ evaluation. Therefore, such a design may overestimate diagnostic accuracy compared to prospective studies enrolling consecutive patients, as it does not fully reflect the natural disease prevalence and clinical spectrum encountered in routine practice. Additionally, the predetermined proportions of MTB, NTM and negative samples could introduce spectrum bias, potentially inflating sensitivity and specificity estimates. Third, our study was conducted at a single tertiary center in Korea, with a sample distribution that may not fully represent the broader global epidemiology of MTB and NTM infections. Regional differences in strain prevalence, patient demographics, and clinical practice patterns could influence the generalizability of the results. To enhance external validity, multi-center studies involving diverse populations and geographic regions are needed. Despite these limitations, our study provides important preliminary evidence supporting the diagnostic potential of the evaluated assays in a real-world clinical setting.

## 5. Conclusions

In conclusion, our study evaluated the diagnostic performance of the PowerChek^TM^ MTB/NTM Real-Time PCR kit Ver.1.0 for the detection of MTB and NTM using clinical specimens, with MGIT culture results as the reference standard. Both real-time PCR assays demonstrated excellent diagnostic performance, achieving 100% sensitivity for MTB and NTM detection. For MTB detection, AdvanSure^TM^ demonstrated a specificity of 100%, whereas a PowerChek^TM^ showed a specificity of 98.62%. For NTM detection, both AdvanSure^TM^ and PowerChek^TM^ exhibited identical specificity of 99.58% for both assays, highlighting their clinical reliability for rapid mycobacterial diagnosis. In addition, our discrepant analysis revealed that the PowerChek^TM^ assay detected four cases of MTB+NTM, which were confirmed as only NTM infections. However, one false-positive result was observed in both PCR kits (NTM) which was confirmed as a negative infection, indicating that further optimization of assay cutoff thresholds or amplification parameters may enhance specificity and reduce diagnostic ambiguity. Additionally, sequence confirmation of a PCR-positive, culture-negative case emphasized the limited sensitivity of culture methods and the complementary value of molecular diagnostics. Overall, both the AdvanSure^TM^ and PowerChek^TM^ real-time PCR assays provide strong, accurate, and clinically valuable tools for the detection of MTB and NTM infections. Their high diagnostic accuracy supports their implementation in routine clinical practice to facilitate early and appropriate management. Nevertheless, in cases of discordant results, confirmatory testing using sequence analysis remains essential. Future studies would be helpful, focusing on further assay refinement, larger-scale validation across diverse clinical settings, and the standardization of diagnostic thresholds to improve performance and minimize false-positive rates.

## Figures and Tables

**Table 1:** Comparison of main specifications of AdvanSure^TM^ TB/NTM and Kogene PowerChek^TM^ MTB/NTM Real-Time PCR kits.

Specification	AdvanSure^TM^ TB/NTM Real-Time PCR Kit	Kogene PowerChek^TM^ MTB/NTM Real-Time PCR Kit
Target pathogens	MTB, NTM	MTB, NTM
Technology	TaqMan chemistry with real-time PCR (SLAN-96P system)	Quadruplex real-time PCR; FAM: ITS, VIC: IS6110, ROX: IC, Cy5: DPC (CFX96^TM^ Dx Real-Time PCR System)
Target genes	IS6110 (MTB), ITS (NTM)	IS6110 (MTB), ITS (NTM)
Specimen type	EDTA-whole blood, tissue, CSF, sputum, bronchoalveolar lavage (BAL), other respiratory specimens, body fluid	Sputum, BAL, other respiratory specimens, body fluid
Sample volume for DNA extraction	200 μL	200 μL
Template volume for PCR	5 μL	5 μL
Total reaction volume	25 μL	20 μL
PCR run time	Approximately 2 h	Approximately 1 h 30 min
Storage Conditions	PRE-Kit: 1–30 °C AMP Kit: −25 to −15 °C (Avoid repeated freeze–thaw)	−20 °C (Avoid repeated freeze–thaw)
Throughput	96 tests/kit	96 tests/kit
Estimated cost per one test (KRW)	14,792	14,000

Note: MTB—*Mycobacterium tuberculosis*; NTM—nontuberculous mycobacteria; IS6110—a gene sequence specific to *mycobacterium tuberculosis*; ITS—internal transcribed spacer, a gene region used for identifying nontuberculous mycobacteria; FAM—fluorescein amidite, a fluorophore used in real-time PCR detection; VIC—a fluorophore used in real-time PCR detection; ROX—a passive reference dye used in real-time PCR to normalize fluorescence; Cy5—a fluorophore used in real-time PCR detection; DPC—diagnostic positive control; EDTA—ethylenediaminetetraacetic acid; CSF—cerebrospinal fluid; BAL—bronchoalveolar lavage; PRE-Kit—Pre-PCR kit; AMP Kit—amplification kit; PCR—polymerase chain reaction; TaqMan—a technology used in PCR for detecting DNA; SLAN-96P—a PCR system used in AdvanSure^TM^; CFX96^TM^ Dx—a PCR system used in PowerChek^TM^; DNA—deoxyribonucleic acid.

**Table 2:** Diagnostic performance of AdvanSure^TM^ TB/NTM and PowerChek^TM^ MTB/NTM Real-time PCR Kits.

Kit	Target	TP	FP	FN	TN	Sensitivity % (95% C.I.)	Specificity % (95% C.I.)	PPV % (95% C.I.)	NPV % (95% C.I.)	Accuracy % (95% C.I.)
AdvanSure^TM^	MTB	100	0	0	290	100 (96.38–100)	100 (98.74–100)	100 (96.38–100)	100 (98.74–100)	100 (99.06–100)
	NTM	150	1	0	239	100 (97.57–100)	99.58 (97.70–99.99)	99.34 (95.50–99.91)	100 (98.47–100)	99.74 (98.58–99.99)
Power Chek^TM^	MTB	100	4	0	286	100 (96.38–100)	98.62 (96.51–99.62)	96.15 (90.43–98.51)	100 (98.72–100)	98.97 (97.39–99.72)
	NTM	150	1	0	239	100 (97.57–100)	99.58 (97.70–99.99)	99.34 (95.50–99.91)	100 (98.47–100)	99.74 (98.58–99.99)

Note: MTB—*Mycobacterium tuberculosis*; NTM—nontuberculous mycobacteria; NG—no growth; PCR—polymerase chain reaction; TP—true positive; TN—true negative; FP—false positive; FN—false negative; CI—confidence interval; PPV—positive predictive value; NPV—negative predictive value; AdvanSure^TM^—AdvanSure^TM^ TB/NTM Real-time PCR kit; PowerChek^TM^—PowerChek^TM^ MTB/NTM Real-time PCR kit Ver.1.0. Diagnostic performance of the real-time PCR assays, including sensitivity, specificity, PPV, NPV, accuracy, and 95% CIs, was calculated using MedCalc Statistical Software (MedCalc Soft-ware Ltd., Ostend, Belgium).

**Table 3:** Discrepant results between AdvanSure^TM^ TB/NTM Real-Time PCR Kit and Kogene PowerChek^TM^ MTB/NTM Real-Time PCR Kits.

Serial No.	AdvanSure^TM^ TB/NTM	PowerChek^TM^ MTB/NTM	MGIT Culture Result	Sequence Analysis	Final Diagnosis *
NTM010	NTM	MTB+NTM	NTM	NTM	**NTM infection**
NTM013	NTM	MTB+NTM	NTM	NTM	**NTM infection**
NTM117	NTM	MTB+NTM	NTM	MTB+NTM	**NTM infection**
NTM135	NTM	MTB+NTM	NTM	NTM	**NTM infection**
NG035	NTM	NTM	Negative	NTM	**Negative**

Note: Discrepant results between the AdvanSure^TM^ TB/NTM and the PowerChek^TM^ MTB/NTM Real-Time PCR Kits were further evaluated through base sequence analysis. * Final diagnosis is confirmed through electronic medical chart review and culture result. Mycobacteria Growth Indicator Tube (MGIT) culture results are included for additional reference. In this context, MTB refers to *Mycobacterium tuberculosis*, NTM to nontuberculous mycobacteria, and NG indicates specimens with no mycobacterial growth.

## Data Availability

The datasets used during the study are available from the corresponding author upon a reasonable request.

## Abbreviations

The following abbreviations are used in this manuscript:

AdvanSure^TM^	AdvanSure^TM^ TB/NTM Real-Time PCR kit
AMP Kit	Amplification kit
BAL	Bronchoalveolar lavage
CI	Confidence interval
CSF	Cerebrospinal fluid
Cy5	A fluorophore used in real-time PCR detection
DNA	Deoxyribonucleic acid
DPC	Diagnostic positive control
EDTA	Ethylenediaminetetraacetic acid
FAM	Fluorescein amidite, a fluorophore used in real-time PCR detection
FN	False negative
FP	False positive
IS6110	A gene sequence specific to Mycobacterium tuberculosis
ITS	Internal transcribed spacer, a gene region used for identifying nontuberculous mycobacteria
MTB	Mycobacterium tuberculosis
NG	No growth
NPV	Negative predictive value
NTM	Nontuberculous mycobacteria
PCR	Polymerase chain reaction
PPV	Positive predictive value
PowerChek^TM^	PowerChek^TM^ MTB/NTM Real-Time PCR kit Ver.1.0
PRE-Kit	Pre-PCR kit
ROX	A passive reference dye used in real-time PCR to normalize fluorescence
SLAN-96P	A PCR system used in AdvanSure^TM^
TaqMan	A technology used in PCR for detecting DNA
TN	True negative
TP	True positive
VIC	A fluorophore used in real-time PCR detection
